# Development of an intravital imaging system for the synovial tissue reveals the dynamics of CTLA-4 Ig in vivo

**DOI:** 10.1038/s41598-020-70488-y

**Published:** 2020-08-10

**Authors:** Tetsuo Hasegawa, Junichi Kikuta, Takao Sudo, Erika Yamashita, Shigeto Seno, Tsutomu Takeuchi, Masaru Ishii

**Affiliations:** 1 Department of Immunology and Cell Biology, Graduate School of Medicine and Frontier Biosciences, Osaka University, 2-2 Yamada-oka, Suita, Osaka 565-0871 Japan; 2 Division of Rheumatology, Department of Internal Medicine, Keio University School of Medicine, Tokyo, Japan; 3 WPI-Immunology Frontier Research Center, Osaka University, Osaka, Japan; 4 Department of Bioinformatic Engineering, Graduate School of Information Science & Technology, Osaka University, Osaka, Japan

**Keywords:** Biological techniques, Rheumatology

## Abstract

There have been many attempts to visualize the inflamed joints using multiphoton microscopy. However, due to the hypervascular and multilayered structure of the inflamed synovium, intravital imaging of the deep synovial tissue has been difficult. Here, we established original intravital imaging systems to visualize synovial tissue and pathological osteoclasts at the pannus–bone interface using multiphoton microscopy. Combined with fluorescence-labeling of CTLA-4 Ig, a biological agent used for the treatment of rheumatoid arthritis, we identified that CTLA-4 Ig was distributed predominantly within the inflamed synovium and bound to CX_3_CR1^+^ macrophages and CD140a^+^ fibroblasts 6 h after injection, but not to mature osteoclasts. Intravital imaging of blood and lymphatic vessels in the inflamed synovium further showed that extravasated CTLA-4 Ig was immediately drained through lymphatic vessels under acute arthritic conditions, but the drainage activity was retarded under chronic conditions. These results indicate that this intravital synovial imaging system can serve as a platform for exploring the dynamics of immune cells, osteoclasts, and biological agents within the synovial microenvironment in vivo.

## Introduction

Rheumatoid arthritis (RA) is one of the most common autoimmune diseases, affecting approximately 1% of the world’s population. Persistent inflammation in the hypertrophied synovium (called “pannus”) leads to the development of pathological osteoclasts on the outer surface of the articular bone, eventually resulting in permanent disability^1^. Over the past two decades, the development of biological agents targeting tumor necrosis factor (TNF), interleukin (IL)-6, and B7 ligands has substantially improved the clinical outcome of RA patients.

CTLA-4 Ig (Abatacept) is a fusion protein of human CTLA-4 and the Fc portion of human IgG1, which binds to B7 ligands expressed by macrophages and dendritic cells to inhibit costimulation of naïve CD4 T cells^2^ and prevents the development of arthritis in animal models^3^. However, CTLA-4 Ig treatment was also shown to be effective in treating established arthritis in collagen-induced arthritis (CIA) mouse model^4^ and human RA patients, in whom memory T cells predominate over naïve T cells in the inflamed synovium^5^; these memory T cells are much less dependent on costimulatory pathways compared to naïve T cells. In addition, CTLA-4 Ig alleviated disease activity in the absence of CD4^+^ T cells in a CIA model^6^, and a number of approaches targeting T cells, including anti-CD4 antibody^7^, have shown no clear benefits in clinical trials of RA patients, suggesting that the action of CTLA-4 Ig may not depend solely on the T cell costimulatory pathway. Indeed, several studies using bone marrow (BM) cells elucidated the mechanism of action of CTLA-4 Ig against antigen-presenting cells through reverse signaling, with induction of tryptophan catabolism^8^. Other studies indicated that CTLA-4 Ig targets endothelial cells to induce immune modulation^9^. Taken together, these reports imply that the mode of action of CTLA-4 Ig is diverse. However, empirical evidence regarding the cells to which CTLA-4 Ig actually binds in vivo is still lacking, and this information is important when designing experiments exploring the mechanism of action of drugs.

In this study, we developed an intravital imaging system using multiphoton microscopy to directly visualize immune cells, pathological osteoclasts, and CTLA-4 Ig in the inflamed synovium. We combined this system with flow cytometry (FCM) to investigate the dynamics and target cells of CTLA-4 Ig within the synovial microenvironment under arthritic conditions.

## Results

### Establishment of intravital imaging system for pathological osteoclasts at the pannus–bone interface

**Figure 1:**
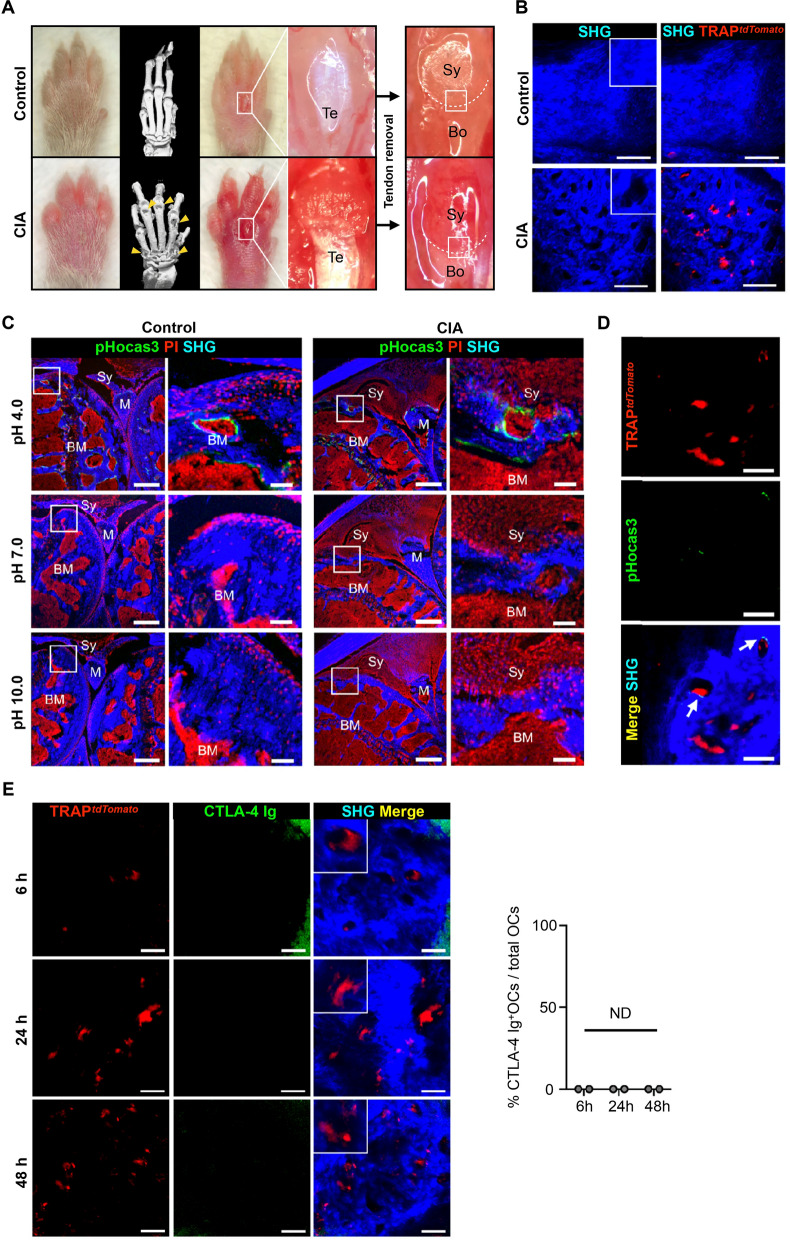
Establishment of intravital imaging system of mature osteoclasts at the pannus–bone interface. (**A**) Pictures and micro-computed tomography (CT) images showing the protocol for imaging of the pannus–bone interface. Arrowheads indicate the erosive area of the joints. After incision of the skin and the extensor tendon of the middle digit, the third meta phalangeal joint of the forepaw was exposed under a stereoscopic microscope. The boxed areas indicate the region of interest. *Te* tendon, *Bo* bone, *Sy* synovium. (**B**) Intravital images of the third meta phalangeal joint of TRAP-tdTomato transgenic mice under control and CIA conditions 2 weeks after the onset of arthritis. Images are representative of at least three independent experiments with similar results. Bars, 100 μm. (**C**) Histological images of the knee joints in control and CIA mice after 3 days of pHocas-3 injection. Tissue sections were exposed to buffer solution at pH 4.0, 7.0, and 10.0. BM: bone marrow; *Sy* synovium, *M* meniscus; *SHG* second harmonic generation; *PI* propidium iodide. Bars, 300 (left) and 50 μm (right). (**D**) Intravital images of the third meta phalangeal joint of the CIA TRAP-tdTomato transgenic mice after 3 days of pHocas-3 injection. Arrows indicate bone-resorbing osteoclasts. Images are representative of at least two independent experiments with similar results. Bars, 50 μm. (**E**) Intravital images of the third meta phalangeal joint of the CIA TRAP-tdTomato transgenic mice after injection of 200 μg of CTLA-4 Ig (AF647) at the indicated time points. Percentages of CTLA-4 Ig-binding osteoclasts among all osteoclasts were calculated. *n* = 2 mice for each group. ND: not detected. Bars, 50 μm.

We initially labeled CTLA-4 Ig with Alexa Fluor 647 dye (AF647), which is optimized for in vivo imaging by controlling the ratio of dye to protein, to track the temporal distribution of CTLA-4 Ig in vivo. Previous studies reported that osteoclasts express B7^10^ and CTLA-4 Ig suppresses osteoclastogenesis^8^, but it remains unclear whether CTLA-4 Ig directly binds to and exerts its effect against mature osteoclasts formed at the pannus-bone interface. To address this issue, we developed an intravital imaging system for the pannus–bone interface to simultaneously visualize pathological osteoclasts and CTLA-4 Ig. As the inflamed synovium is a thick, multilayered tissue with a hypervascular structure, light can be easily scattered and intravital imaging of the deep synovial tissue has proven difficult. Therefore, we decided to expose the third meta phalangeal joint of the forepaw, which is much smaller than the ankle joint (as reported on previously)^11,12^ and thin enough to permit penetration of near-infrared excitation laser into the pannus–bone interface. We observed CIA mice 2 weeks after the onset of arthritis, because osteoclast formation and bone destruction occur after continuous inflammation of the synovial tissue. After incision of the skin and the extensor tendon of the middle digit using microscissors under a stereoscopic microscope, the “bare area”, wherein bone is attached to the synovium without a cartilaginous covering, can be directly visualized (Fig. 1A). Bone tissue was visualized by a nonlinear optical process (SHG: second harmonic generation), and small resorption pits (diameter, 20–50 μm) were observed in CIA mice (Fig. 1B). Mature osteoclasts were labeled in TRAP-tdTomato transgenic mice in the DBA/1J background, and some of the resorption pits were covered by mature osteoclasts (Fig. 1B right panel). To further confirm that these osteoclasts at the pannus–bone interface were actively resorbing the bone matrix, we injected the pH-sensing chemical probe (pHocas-3)^13^, which was previously developed to distribute to the bone surface and emit fluorescence in the acidic region, thereby allowing identification of actively bone-resorbing osteoclasts in living animals. Histological examination at varying pH levels showed that the pHocas3 fluorescence was detected on the bone surface of the BM under the control condition and on both the BM and pannus–bone interface of arthritic knee joints under acidic conditions (Fig. 1C). Therefore, we examined the erosive region in vivo using TRAP-tdTomato transgenic mice, and the results indicated that some of the mature osteoclasts were actively resorbing the bone (Fig. 1D). When AF647-labeled CTLA-4 Ig was injected into CIA mice, no merging of tdTomato and AF647 signals was observed in 6, 24, or 48 h after injection of CLTA-4 Ig (Fig. 1E), indicating that CTLA-4 Ig did not bind directly to the pathological osteoclasts at the pannus–bone interface.

### Intravital synovial imaging revealed binding of CTLA-4 Ig with CX_3_CR1^+^ macrophages

**Figure 2:**
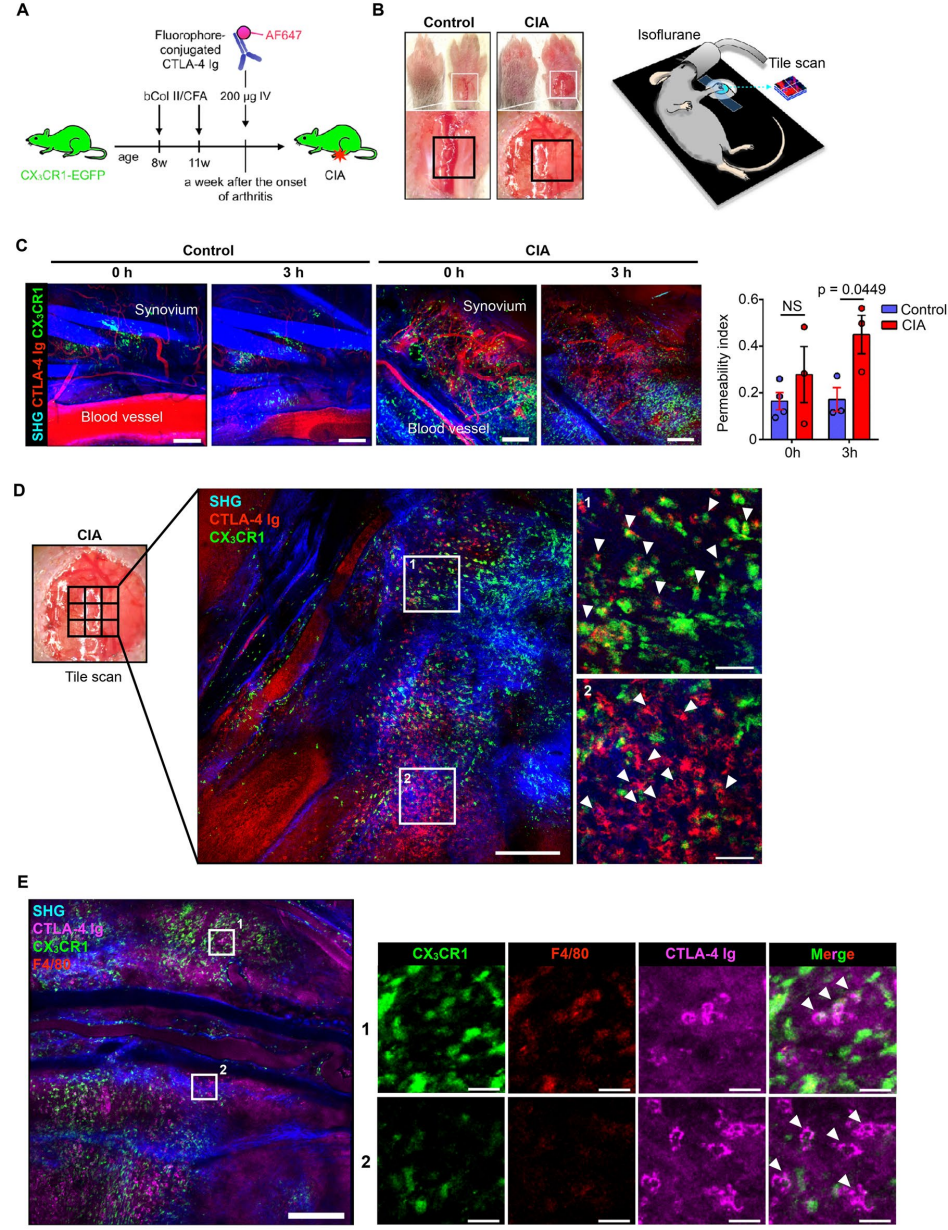
Intravital imaging of the inflamed synovium showing CTLA-4 Ig binding to CX_3_CR1^+^ macrophages. (**A**) Schematic diagram showing the experimental design for multiphoton imaging. (**B**) Area in the wrist joint observed by the inverted multiphoton microscopy. Black squares indicate the fields visualized in (**C**). (**C**) Intravital tile scan images of the synovium of CX_3_CR1 knock-in mice injected with CTLA-4 Ig (AF647) under control or CIA conditions, and their permeability index (described in Supplementary Fig. S1A). Bars, 200 μm. Symbols represent individual mice. (**D**) Intravital tile scan images of the CIA synovium of CX_3_CR1 knock-in mice injected with CTLA-4 Ig (AF647). In the high-power field, SHG signals were weakened to clearly display green and red signals. Arrowheads indicate CTLA-4 Ig accumulation. Images are representative of at least three independent experiments with similar results. Bars, 300 (left) and 50 μm (right). (**E**) Intravital tile scan images of the CIA synovium of CX_3_CR1 knock-in mice injected with CTLA-4 Ig (AF647) and anti-F4/80-phycoerythrin (PE) antibody. Arrowheads indicate CTLA-4 Ig accumulation. Bars, 300 (left) and 30 μm (right). Unpaired two-tailed *t* test (**C**). Mean ± S.E.M. for each group.

As our previous study showed that synovial CX_3_CR1^+^ macrophages express both of the costimulation markers, CD80 and CD86^14^, we used CX_3_CR1-EGFP transgenic mice in the DBA/1J background to visualize the interaction of CTLA-4 Ig and EGFP^+^ cells. CTLA-4 Ig was injected into CIA mice^4^ (Fig. 2A), and we established an intravital imaging protocol to obtain a wide field of view of the inflamed synovium. To minimize the respiratory fluctuation of the organ, we applied the inverted microscopy to fix the wrist joint to the cover glass, and the inflamed tenosynovium was visualized directly with minimal invasion of the skin incision (Fig. 2B). After observing that CTLA-4 Ig accumulated in the inflamed synovial microenvironment, we calculated the permeability index, given by the ratio between the mean fluorescence intensity (MFI) of AF647 in the synovial interstitium and that in the blood vessels, as described previously^15^ (Supplementary Fig. S1A). The permeability index was higher under the CIA condition compared to the control condition, indicating that inflamed conditions accelerates CTLA-4 delivery into the synovium (Fig. 2C). We confirmed the binding of CTLA-4 Ig to CX_3_CR1^+^ cells and also observed binding to CX_3_CR1^−^ cells in the inflamed synovium (Fig. 2D). Because the previous study showed the presence of tissue-resident CX_3_CR1^+^ macrophages and CX_3_CR1^−^ macrophages^16^, we performed intravital double staining of CX_3_CR1 and F4/80 by injecting anti-F4/80-phycoerythrin (PE) antibody into CX_3_CR1-EGFP transgenic mice. This showed that CTLA-4 Ig bound to CX_3_CR1^+^F4/80^+^ (Fig. 2E-1) and CX_3_CR1^−^F4/80^−^ cells (Fig. 2E-2), indicating that CTLA-4 Ig-bound CX_3_CR1^−^ cells were not the CX_3_CR1^−^ macrophages.

### CTLA-4 Ig was distributed predominantly in the inflamed synovium and bound to CX_3_CR1^+^ macrophages and CD140a^+^ fibroblasts

**Figure 3:**
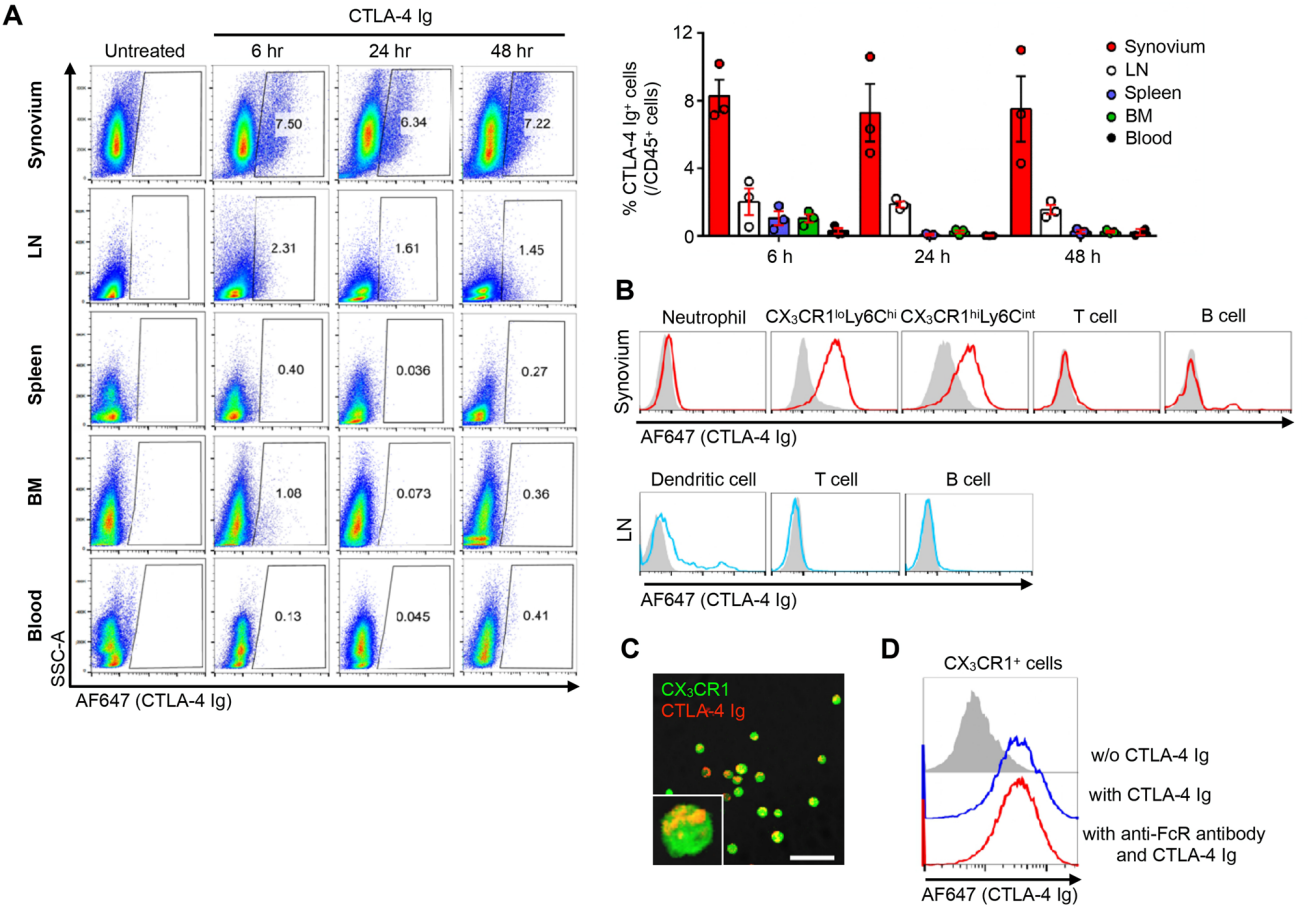
CTLA-4 Ig was predominantly distributed in the inflamed synovium and bound to CX_3_CR1^+^ cells. (**A**) Flow cytometry (FCM) plots and cumulative data of CTLA-4 Ig (AF647)^+^ cells among CD45^+^ cells in the inflamed synovium, lymph node (LN), spleen, bone marrow (BM), and blood. *n* = 3 mice for each group. (**B**) Histogram plots of CTLA-4 Ig (AF647) fluorescence in Ly6G^+^ neutrophils, CX_3_CR1^lo^Ly6C^hi^ cells, CX_3_CR1^hi^Ly6C^int^ cells, CD3^+^T lymphocytes, and CD19^+^ B lymphocytes in the inflamed synovium, and CD11c^+^ dendritic cells, T and B lymphocytes in the draining lymph node (LN). (**C**) Confocal image of CX_3_CR1-EGFP^+^ cells isolated from the inflamed synovium of CX_3_CR1-EGPP knock-in mouse treated with 200 μg CTLA-4 Ig (AF647) 24 h before sacrifice. Bar, 30 μm. (**D**) Histogram plots of CTLA-4 Ig (AF647) fluorescence in CX_3_CR1-EGFP^+^ cells from the inflamed synovium. The FCM-sorted CX_3_CR1-EGFP^+^ cells were treated without CTLA-4 Ig (shaded region), with CTLA-4 Ig, or with CTLA-4 Ig after pretreatment with anti-Fc receptor (FcR) antibody in vitro.

To further elucidate the target populations of CTLA-4 Ig under arthritic conditions, we performed flow cytometry (FCM) analysis in the synovium, lymph nodes, spleen, BM, and blood. Among CD45^+^ cells, CTLA-4 Ig bound prominently to the cells in the synovium 6 h after the CTLA-4 Ig injection, but not in other organs (Fig. 3A). Consistent with the intravital imaging data shown in Fig. 2D and the previous study showing that two monocyte/macrophage lineage populations of synovial CX_3_CR1-EGFP^+^ cells in a CIA model (CX_3_CR1^lo^Ly6C^hi^ and CX_3_CR1^hi^Ly6C^int^ cells) both expressed CD80/CD86^14^, CTLA-4 Ig bound to CX_3_CR1^+^ cells (Fig. 3B). On the other hand, CTLA-4 did not bind to B7^−^ cells, such as T cells and neutrophils, supporting that fluorescent labeling of CTLA-4 Ig did not interfere with its binding specificity. CTLA-4 Ig also partially bound to dendritic cells in the draining lymph nodes (Fig. 3B). Observation of FCM-sorted CX_3_CR1-EGFP^+^ cells by confocal microscopy revealed CTLA-4 Ig on the surface of EGFP^+^ cells (Fig. 3C), which was not disrupted by pretreatment with an anti-Fc receptor (FcR) antibody (Fig. 3D); this indicated that CLTA-4 Ig bound to EGFP^+^ cells through CTLA-4, and not via the Fc portion of the antibody.

**Figure 4:**
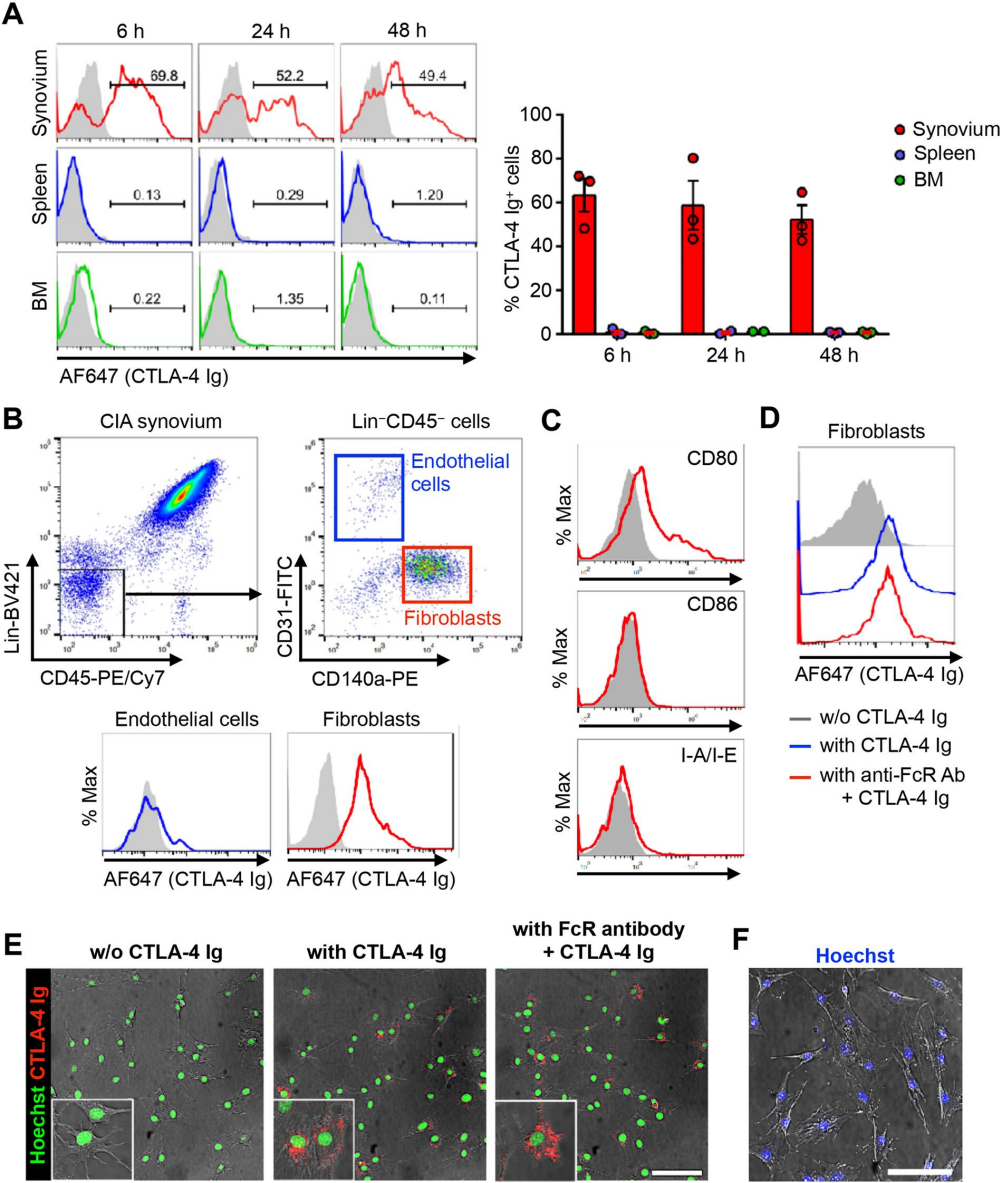
CTLA-4 Ig bound to CD140a^+^ fibroblasts in the inflamed synovium. (**A**) Histograms and cumulative data of CTLA-4 Ig (AF647)^+^ cells among CD45^−^Lin^−^ cells in the inflamed synovium, spleen, and BM. *n* = 3 mice for each group except *n* = 2 mice for the BM group at 24 h. (**B**) Gating strategy for endothelial cells (Lin^−^CD45^−^CD31^+^CD140a^−^ cells) and fibroblasts (Lin^−^CD45^−^CD140a^+^CD31^−^ cells) in the inflamed synovium of CIA mice. Histogram plots of CTLA-4 Ig (AF647) fluorescence in endothelial cells and fibroblasts. Shaded regions indicate cells from an untreated CIA mouse. Data are representative of at least three independent experiments. (**C**) Histogram plots of indicated surface markers in synovial fibroblasts from CIA mice. Shaded regions indicate staining with isotype control. (**D**) Histogram plots of CTLA-4 Ig (AF647) fluorescence in CD140a^+^ fibroblasts from the inflamed synovium. FCM-sorted fibroblasts were treated without CTLA-4 Ig (shaded region), with CTLA-4 Ig, or with CTLA-4 Ig after pretreatment with anti-FcR antibody in vitro. (**E**,**F**) Confocal images of CD140a^+^ fibroblasts isolated from the inflamed synovium of a CIA mouse. (**E**) FCM-sorted fibroblasts were treated without CTLA-4 Ig, with CTLA-4 Ig, or with CTLA-4 Ig after pretreatment with anti-FcR antibody in vitro. (**F**) FCM-sorted fibroblasts on Hoechst and transmission images. Bars, 100 μm.

Because CTLA-4 Ig-bound CX_3_CR1-EGFP^−^ cells (Fig. 2D) was not detected among CD45^+^ cells in the inflamed synovium, we next analyzed CD45^−^ cells in the synovium, spleen, and BM. Consistent with the results for CD45^+^ cells, CTLA-4 Ig bound predominantly to CD45^−^ cells in the inflamed synovium (Fig. 4A), and further classification of CD45^−^ cells into CD45^−^Lin^−^CD31^+^CD140a^−^ cells (endothelial cells) and CD45^−^Lin^−^CD31^−^CD140a^+^ cells (fibroblasts)^14^ revealed that CTLA-4 Ig bound to the synovial fibroblasts (Fig. 4B). In comparison, control IgG labeled with AF647 did not bind to CD45^+^ cells, CD45^−^ cells, or osteoclasts, supporting the specificity of CTLA-4 Ig binding capacity (Supplementary Fig. S2A–D). These CD140a^+^ fibroblasts expressed CD80, but not CD86 or I-A/I-E (Fig. 4C). Pretreatment with anti-FcR antibody did not interfere with the binding of FCM-sorted fibroblasts and CTLA-4 Ig in vitro, as determined by both FCM analysis (Fig. 4D) and confocal microscopy (Fig. 4E). Confocal imaging also confirmed that FCM-sorted CD140a^+^ cells had multipolar and elongated shapes, which was consistent with the morphology of fibroblasts (Fig. 4F). To determine whether reverse signaling was induced by CTLA-4 Ig binding, quantitative real-time PCR of representative fibroblast effector cytokines in arthritis^17^ was performed in the synovial fibroblasts. In vitro CTLA-4 Ig challenge did not alter the expression of any of the effector cytokines, including *M-csf*, *Rankl*, *Il-6*, *Ccl2*, *Ccl3*, *Ccl4*, *Ccl5*, *Cxcl1*, *Cxcl5,* and *Ido* (Supplementary Fig. S3A). Injection of CTLA-4 Ig in vivo also failed to upregulate these cytokines (Supplementary Fig. S3B), indicating that there was no reverse signaling in the synovial fibroblasts.

### Extravasated CTLA-4 Ig was immediately drained by lymphatic vessels in the inflamed synovium in acute CIA

**Figure 5:**
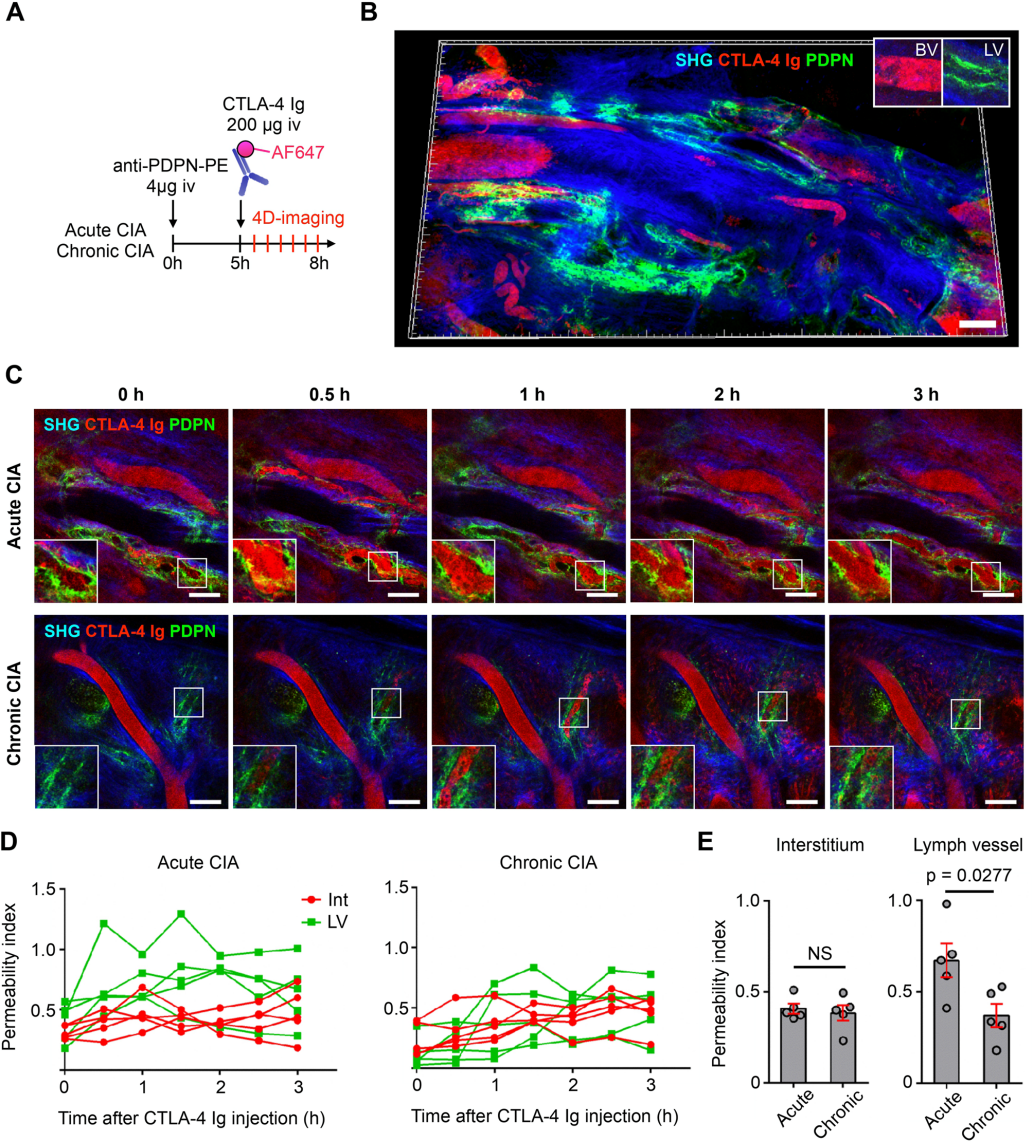
Extravasated CTLA-4 Ig was immediately drained by synovial lymphatic vessels in acute CIA. (**A**) Schematic diagram showing the experimental design for multiphoton imaging of acute CIA (3 days after the onset of arthritis) and chronic CIA (3 weeks after the onset of arthritis). Lymphatic endothelial cells were stained by intravenous injection of anti-podoplanin (PDPN)-PE antibody 5 h before the imaging. (**B**) Intravital tile scan image of the synovium of acute CIA mice immediately after injection of CTLA-4 Ig (AF647). *BV* blood vessel, *LV* lymphatic vessel. Images are representative of at least three independent experiments with similar results. Bar, 200 μm. (**C**) Intravital images of the inflamed synovium at the indicated time points after injection of CTLA-4 Ig (AF647). (**D**) Mean fluorescence intensities of AF647 in the BV, interstitium (Int), and LV area were measured to calculate permeability index in each time point with Imaris software. The average of the permeability index for 3 h was shown in (**E**). Unpaired two-tailed *t* test. Mean ± S.E.M. for each group. Symbols represent individual visual fields compiled from three mice for each group.

We next investigated how the extravasated CTLA-4 Ig was cleared from the interstitium of the synovium under arthritic conditions. Lymphatic endothelial cells were labeled by intravenous injection of phycoerythrin (PE)-conjugated antibody against podoplanin (anti-PDPN-PE), which is expressed specifically in the lymphatic endothelium but not in the blood vasculature. CTLA-4 Ig (AF647) was injected 5 h after anti-PDPN-PE injection (Fig. 5A), and blood and lymphatic vessels were clearly visualized in vivo using this protocol (Fig. 5B). When CTLA-4 Ig was injected 3 days after the onset of arthritis, CTLA-4 Ig showed extravasation and started to drain via the lymphatic vessels immediately after injection. On the other hand, when CTLA-4 Ig was injected 3 weeks after the onset of arthritis, lymphatic drainage of CTLA-4 Ig was reduced (Fig. 5C and D). Although the mean permeability index of the 3-h observation was comparable between acute and chronic CIA mice in the interstitial space, that in lymphatic vessels was higher in acute CIA mice. These results indicated that the drainage capacity of lymphatic vessels in the synovium differs between acute and chronic arthritic conditions, which may affect the dynamics of CTLA-4 Ig within the synovial microenvironment.

## Discussion

We have developed an original protocol for real-time intravital imaging of the inflamed synovium and mature osteoclasts at the pannus–bone interface by multiphoton microscopy. Although there have been many important attempts to visualize inflamed joints using multiphoton microscopy^11,12,18,19^, there were still several obstacles to obtaining a wide field of view of synovial tissues and mature osteoclasts formed at the pannus–bone interface in vivo. First, abundant red blood cells in the hypervascular inflamed synovium readily scatter light, thereby impeding deep tissue imaging, such as of the pannus–bone interface. Microbleeding from the inflamed joint with invasive procedures can also prevent the emitted photons from reaching the objective of the microscope. Second, the hypertrophied synovial tissue is a multilayered structure composed of synovial lining and sublining cells, blood vessels, lymphatic vessels, and fibrous tissues. Although multiphoton microscopy uses light of near-infrared wavelengths, which can penetrate more than 100 μm into tissues^20^, the complex multilayered structure of the inflamed synovium with heterogeneous refraction factors limits the depth of observation. Third, intravital imaging with upright multiphoton microscopy is relatively more vulnerable to drift of visual field due to respiratory movement than is inverted microscopy, where the region of interest is fixed directly to the cover glass and large visual fields can be obtained. To overcome these issues, we decided to expose a smaller joint than the ankle joint and performed intravital imaging with the inverted microscopy. All procedures were performed with microscissors under a stereoscopic microscope to minimize microbleeding. As a result, we achieved a wide field of view of the wrist joints by applying tile scan and observed the living pathological osteoclasts resorbing bone at the pannus–bone interface for the first time. A recent important study visualized the interaction of CD4 T cells and dendritic cells using a model induced by periarticular injection of heat-aggregated ovalbumin and adoptive transfer of OT-II cells^12^. Although previous studies observed the joint tissue after the periarticular injection of inflammation-inducing substances or pathogens,^12,18,19^ which may affect the periarticular and subcutaneous tissue, our protocol can be applied to the autoimmune-induced arthritis model, CIA, which shows synovitis and arthritic bone erosion.

The binding of CTLA-4 Ig to CX_3_CR1^+^ cells was consistent with our previous observation that both CX_3_CR1^lo^Ly6C^hi^ and CX_3_CR1^hi^Ly6C^int^ cells, the highly osteoclastogenic macrophages in the joint, express CD80/CD86^14^. This supported the significant study showing effects of CTLA-4 Ig against osteoclastogenesis^8^. On the other hand, binding of CTLA-4 Ig to synovial fibroblasts has not been reported. A previous study showed that interferon gamma (IFN-γ) plus TNF induce expression of CD80, but not CD86, on fibroblasts^21^, in turn suggesting that CD80 expression on synovial fibroblasts may be dependent on the inflammatory milieu within the inflamed synovium. As the binding of CD80 and CTLA-4 is strong^22,23^ and potent^24^, synovial fibroblasts can be one of the targets for CTLA-4 Ig in arthritis. However, it remains unclear whether CTLA-4 Ig exerts any activity against synovial fibroblasts, or whether the interaction of CD28 and CD80 on fibroblasts has any biological significance. As CD80 was reported to activate natural killer cells in an MHCII-independent manner^25^, and to enhance T cell proliferation^21^, further studies are needed to elucidate the biological function of CD80 in synovial fibroblasts. CTLA-4 Ig also partially bound to dendritic cells in the draining lymph nodes, suggesting that they can function in inhibiting antigen presentation.

Taking advantage of our intravital imaging system for the synovium and the chronicity of the CIA model, we further examined the synovial lymphatic system in vivo, which is one of the under-recognized fields. Our results showed that intravenously injected CTLA-4 Ig was immediately distributed to the interstitium and drained through lymphatic vessels under conditions of acute arthritis, while the drainage activity was partially disrupted under chronic conditions. These observations suggest reduced drainage of inflammatory cells and cytokines in chronic arthritis, contributing to the prolonged inflammation and chronicity in RA. Lymphatic contraction is negatively regulated through cytokine-mediated nitric oxide (NO) signaling^26^, and direct application of TNF decreases lymphatic contraction *ex vivo*^27^. In addition, ultrasonography power Doppler examination revealed collapsed draining lymph nodes during chronic synovitis^26,28^. These studies indicated that prolonged inflammatory cytokine exposure and collapsed draining lymph nodes in chronic arthritis may directly and indirectly affect lymphatic flow, thereby limiting drainage capacity from the synovium and drug delivery to the draining lymph nodes. Lymphatic drainage from inflamed joints has been used to serve as biomarkers of arthritis and the response to the therapy^29,30^. Thus, early intervention in acute synovitis may prevent the deterioration of the synovial lymphatic system and promote drainage of inflammatory cytokines from the synovium to mitigate disease progression.

Our study has several limitations. First, due to AF647 fluorescence bleaching, CTLA-4 Ig could only be visualized up to 72 h after the injection; therefore, our observation can only be applied to early time points after the drug injection. Second, the observation time point is a study limitation, as the pannus-bone interface cannot be visualized before 2 weeks following the onset of the disease because marked swelling prevents us from exposing the deep tissue without considerable microbleeding.

In conclusion, we developed a real-time intravital imaging system of the inflamed synovium and pathological osteoclasts at the pannus–bone interface using multiphoton microscopy. This system, combined with the antibody labeling strategy, allowed analysis of the distribution and intravital target populations of CTLA-4 Ig within living synovial tissue. In addition, success in visualizing pathological osteoclasts formed in the arthritic joints enables us to compare the intravital behavior of osteoclasts in a physiological BM microenvironment and those in a pathological arthritic environment. These results indicated that our intravital imaging system for the inflamed synovium can serve as a platform for exploring the dynamics of immune cells, pathological osteoclasts, and biological agents in living animals, and should allow unbiased observation of the synovial microenvironment to better understand the pathogenesis of arthritis.

## Methods

### Mice

WT DBA/1J mice were obtained from Oriental Yeast Co. in Japan. CX_3_CR1-EGFP knock-in mice^31^ and TRAP promoter-tdTomato transgenic mice^32^ in the C57BL/6J (B6) background were backcrossed > 10 generations onto DBA/1J mice as reported previously^14^. All mice were bred and maintained under specific pathogen-free conditions at the animal facilities of Osaka University, and all animal experiments were performed in accordance with the Osaka University Animal Experimental Guidelines using approved protocols. All animal studies were approved by the Institutional Animal Care and Use Committee of Osaka University.

### Animal models

Arthritis was induced in DBA/1J mice between 8 and 10 weeks of age, as described previously^33^. Chicken type II collagen (cCII; Sigma Chemical Co, St Louis, Missouri) was dissolved in 0.05 M acetic acid to a concentration of 4.0 mg/ml by overnight rotation at 4 °C and mixed with an equal volume of Freund's complete adjuvant (Wako Pure Chemical Industries, Ltd.). On day 0, DBA/1 J mice were immunized at the base of the tail with 100 μl of emulsion. The same injection was repeated on day 21. For CTLA-4 Ig treatment, mice were injected intravenously with 200 μg of CTLA-4 Ig (Bristol-Myers Squibb) labeled using a SAIVI Alexa Fluor 647 Antibody/Protein-Labeling Kit (S30044, Thermo Fisher Scientific), which can control the degree of labeling to prevent dispersion of the signal-to-background ratio, biodistribution, and clearance. We optimized the labeling conditions to achieve about four to five molecules of dye per antibody. Human IgG isotype control (02-7102, Thermo Fisher Scientific) was used for control experiments.

### Intravital imaging

Mice were anesthetized with isoflurane (Wako Pure Chemical Industries, Ltd.) and the tenosynovium of the dorsal wrist joint was surgically exposed by removing the skin with microscissors under a stereoscopic microscope (SMZ 745 T, Nikon, Tokyo, Japan) as shown in Fig. 2B. For imaging of the third meta phalangeal joint of the forepaw, the skin was incised and the extensor tendon of the middle digit was removed with microscissors under a stereoscopic microscope, as shown in Fig. 1A. The synovium was observed via TCS-SP5 confocal and multiphoton microscope (Leica Microsystems) equipped with a 25 × water-immersion objective. Synovial fibrous tissues and bone were visualized using the second harmonic generation (SHG) with infrared lasers driven by a Chameleon XR Sapphire laser (Coherent). The excitation wavelength of 880 nm was used to excite SHG and EGFP, and 940 nm to excite tdTomato and pHocas-3. The synovium was further illuminated with a laser (wavelength: 488 nm for EGFP, 561 nm for tdTomato, and 633 nm for AF647). Emission signals were obtained by setting the wavelength to 430–480 nm for SHG, 500–550 nm for EGFP, 565–605 nm for tdTomato, and 640–670 nm for AF647.

Image stacks were collected with a vertical step size of 4 − 5 μm to a depth of 50–150 μm below the synovial surface. Imaging data were processed using Imaris software (Bitplane) to create maximum intensity projection (MIP) images with median filters for noise reduction when necessary. Large three-dimensional tile scans of the synovium were acquired by stitching adjacent *z*-stack images with Leica Application Suite AF 2.7.3.9723.

A pH-sensing chemical probe (pHocas-3) dissolved in phosphate-buffered saline (PBS) was injected subcutaneously at 6 mg per kg body weight daily into TRAP-tdTomato transgenic mice in the DBA/1J background for 3 days before imaging^13^.

Permeability was quantified as described previously^15^. Briefly, the blood vessel area, lymphatic vessel area, and interstitial area were manually circumscribed at each time point. The permeability index is defined as the ratio of mean fluorescence intensity (MFI) of AF647 in the synovial interstitial area or lymphatic vessel area to that in the blood vessel area, as described in Supplementary Fig. 1.

### Isolation of cells from tissues

After sacrifice under anesthesia, the right auricles of the mice were cut and 15 ml of pre-warmed 1 × PBS was injected into the left ventricle for perfusion. Perfusion was omitted in experiments designed to assess blood samples. The protocol of isolating the inflamed synovium was described previously^14^. Briefly, mice were perfused with 15 ml of pre-warmed 1 × PBS. After the removal of the skin and biceps femoris muscle, the quadriceps femoris muscles and patellar ligament, including the patella, were removed from the knee joint under a stereoscopic microscope (SMZ 745T; Nikon). Ankle joint tendons, including the Achilles tendon, were removed to reveal the hypertrophied synovium around the talus, which allowed for isolation without damaging the bone. Synovial tissues were digested with 3 mg/ml type I collagenase in Hanks’ balanced salt solution (HBSS), and incubated at 37 °C for 30 min.

The BM, spleen, and draining lymph nodes were minced and passed through a 70-μm cell strainer for FCM analysis.

### Flow cytometry

Flow cytometry analyses were performed as described previously^14^. Briefly, measurements were performed on an SH800 cell sorter (Sony) and analyzed with FlowJo software (TreeStar). Isolated murine cells were blocked with anti-CD16/32 antibody (553141; BD Biosciences; 2.4G2; 8330543) for 10 min, followed by staining with the following antibodies for 15 min: anti-CD80-BV421 (104725; BioLegend; 16-10A1; B264061), anti-CD86-BV421 (105,031; BioLegend; B261490), anti-CD3-BV421 (100227; BioLegend; 17A2; B245756), anti-Ly6G-BV421 (127627; BioLegend; 1A8; B241491), Streptavidin-BV421 (405226; BioLegend; B274676), anti-I-A/I-E-Biotin (107603; BioLegend; M5/114.15.2; B189443), Lineage cell detection cocktail-Biotin (130-092-613; Miltenyi Biotec; 5161024423), anti-CD140a-PE (135905; BioLegend; APA5; B244566), anti-CD45-FITC (103108; BioLegend; 30-F11; B176444), anti-CD45-PE/Cy7 (103114; BioLegend; 30-F11; B271123), anti-Ly6C-PE/Cy7 (128017; BioLegend; HK1.4; B268312), anti-CD19-PE/Cy7 (552854; BD Biosciences; 1D3; 2342827), anti-CD31-FITC (102405; BioLegend; 390; B178358), Biotin-Rat IgG2a,κ isotype control (400503; BioLegend; RTK2758; B227705), BV421-Rat IgG2a,κ isotype control (400535; BioLegend; RTK2758; B259985), BV421-Armenian Hamster IgG isotype control (400935; BioLegend; HTK888; B266305). Anti-F4/80-PE antibody (123110; BioLegend; BM8; B282878) was used for intravital imaging.

### Histology and immunohistochemistry

Immunohistological analyses were performed as described previously^14^. A pH-sensing chemical probe (pHocas-3) dissolved in PBS was injected subcutaneously at 6 mg per kg body weight daily for 3 days before sacrifice. Immediately after sacrifice, the samples were frozen in chilled hexane (Wako) using dry ice, and 12-μm-thick sections of non-decalcified knee joints were prepared using a Multi-Purpose Cryosection Preparation Kit^34^ (Section-Lab and Leica Microsystems). Then, the samples were stained with propidium iodide and observed using a TCS-SP5 confocal microscope (Leica Microsystems). Collagen-enriched bone matrices were visualized using the second harmonic generation (SHG) with infrared lasers of a TCS-SP5 multi-photon laser microscope driven by a Chameleon XR Sapphire laser (Coherent). Technical buffer solutions (Mettler-Toledo) were used to control the pH of the sections.

### Quantitative real-time PCR analyses

Quantitative real-time PCR analyses were performed as described previously^14^. Briefly, total RNA and cDNA of the cells from each tissue were obtained with RNeasy Mini Kit (Qiagen) and Superscript III reverse transcriptase (Thermo Fisher Scientific), according to the manufacturers’ instructions. Quantitative real-time PCR was performed for 40 cycles using a Thermal Cycler Dice Real-Time System TP800 (Takara). The reactions were normalized relative to the housekeeping gene *β-Actin*. The following specific primer pairs for mice were used (forward and reverse, respectively)^35^: *β-actin* (5′-TCCTCCCTGGAGAAGAGCTA-3′ and 5′-ATCTCCTTCTGCATCCTGTC-3′); *Csf-1* (5′-CCCATATTGCGACACCGAA-3′ and 5′-AAGCAGTAACTGAGCAACGGG-3′); *Rankl* (5′-CAGCATCGCTCTGTTCCTGTA-3′ and 5′-CTGCGTTTTCATGGAGTCTCA-3′); *Il − 6* (5′-CACATGTTCTCTGGGAAATCG-3′ and 5′-TTGTATCTCTGGAAGTTTCAGATTGTT-3′); *Ccl2* (5′-GCTCAGCCAGATGCAGTTAAC-3′ and 5′-CTCTCTCTTGAGCTTGGTGAC-3′); *Ccl3* (5′-ACCATGACACTCTGCAACCA-3′ and 5′-CCCAGGTCTCTTTGGAGTCA-3′); *Ccl4* (5′-CCACTTCCTGCTGTTTCTCTTA-3′ and 5′-CTGTCTGCCTCTTTTGGTCAG-3′); *Ccl5* (5′-ATATGGCTCGGACACCACTC-3′ and 5′-TTCCTTCGAGTGACAAACACG-3′); *Cxcl1* (5′-GGCGCCTATCGCCAATG-3′ and 5′-CTGGATGTTCTTGAGGTGAATCC-3′); *Cxcl5* (5′-GCTGCCCCTTCCTCAGTCAT-3′ and 5′-CACCGTAGGGCACTGTGGAC-3′); *Ido* (5′-CGGACTGAGAGGACACAGGTTAC-3′ and 5′-ACACATACGCCATGGTGATGTAC-3′).

### Statistical analysis

The results are shown as single data points in dot plots and as mean ± SEM. Between-group differences were determined using the two-tailed *t* test. Statistical analyses were performed using GraphPad Prism (GraphPad Software Inc.). Animal sample sizes are indicated on each figure legends. In all analyses, *p* < 0.05 was taken to indicate statistical significance.

## Supplementary information

Supplementary Information.

## Data Availability

Data are available from the corresponding author on reasonable request.

## References

[1] Schett G, Gravallese E (2012). Bone erosion in rheumatoid arthritis: Mechanisms, diagnosis and treatment. Nat. Rev. Rheumatol..

[2] Linsley PS (1992). Immunosuppression in vivo by a soluble form of the CTLA-4 T cell activation molecule. Science (80-.).

[3] Knoerzer DB, Karr RW, Schwartz BD, Mengle-Gaw LJ (1995). Collagen-induced arthritis in the BB rat. Prevention of disease by treatment with CTLA-4-Ig. J. Clin. Invest..

[4] Webb LMC, Walmsley MJ (1996). Prevention and amelioration of collagen-induced arthritis by blockade of the CD28 co-stimulatory pathway : requirement for both B7–1 and B7–2. Eur. J. Immunol..

[5] Bluestone JA, Clair EWS, Turka LA, Carolina N (2006). CTLA4Ig: Bridging the basic immunology with clinical application. Immunity.

[6] Jansen DTSL (2015). Abatacept decreases disease activity in a absence of CD4+ T cells in a collagen-induced arthritis model. Arthritis Res. Ther..

[7] Mason U (2002). CD4 coating, but not CD4 depletion, is a predictor of efficacy with primatized monoclonal anti-CD4 treatment of active rheumatoid arthritis. J. Rheumatol..

[8] Bozec A (2014). T cell costimulation molecules CD80/86 inhibit osteoclast differentiation by inducing the IDO/tryptophan pathway. Sci. Transl. Med..

[9] Cutolo M, Nadler SG (2013). Advances in CTLA-4-Ig-mediated modulation of inflammatory cell and immune response activation in rheumatoid arthritis. Autoimmun. Rev..

[10] Li H (2010). Crosstalk between the bone and immune systems: osteoclasts function as antigen-presenting cells and activate CD4+ and CD8+ T cells. Blood.

[11] Byrne R (2012). A dynamic real time in vivo and static Ex vivo analysis of granulomonocytic cell migration in the collagen-induced arthritis model. PLoS ONE.

[12] Prendergast CT (2018). Visualising the interaction of CD4 T cells and DCs in the evolution of inflammatory arthritis. Ann. Rheum. Dis..

[13] Maeda H (2016). Real-Time intravital imaging of pH variation associated with osteoclast activity. Nat. Chem. Biol..

[14] Hasegawa T (2019). Identification of a novel arthritis-associated osteoclast precursor macrophage regulated by FoxM1. Nat. Immunol..

[15] Egawa G (2013). Intravital analysis of vascular permeability in mice using two-photon microscopy. Sci. Rep..

[16] Culemann S (2019). Locally renewing resident synovial macrophages provide a protective barrier for the joint. Nature.

[17] Christopher DB, Filter A (2017). Fibroblasts and fibroblast-like synoviocytes. Kelley Firestein’s Textb. Rheumatol..

[18] Wang B (2012). In vivo imaging implicates CCR2+ monocytes as regulators of neutrophil recruitment during arthritis. Cell Immunol..

[19] Zinselmeyer BH, Lynch JN, Zhang X, Aoshi T, Miller MJ (2008). Video-rate two-photon imaging of mouse footpad—a promising model for studying leukocyte recruitment dynamics during inflammation. Inflamm. Res..

[20] Hasegawa T, Kikuta J, Ishii M (2019). Imaging the bone-immune cell interaction in bone destruction. Front. Immunol..

[21] Pechhold K (1997). Inflammatory cytokines IFN-γ plus TNF-α induce regulated expression of CD80 (B7–1) but not CD86 (B7–2) on murine fibroblasts. J. Immunol..

[22] Thompson CB (1995). Distinct roles for the costimulatory ligands B7–1 and B7–2 in T helper cell differentiation?. Cell.

[23] Stamper CC (2001). Crystal structure of the B7–1/CTLA-4 complex that inhibits human immune responses. Nature.

[24] Yamada A (2001). CD28-independent Costimulation of T cells in alloimmune responses. J. Immunol..

[25] Stambrook PJ, Maher J, Farzaneh F (2017). Cancer immunotherapy: Whence and whither. Mol. Cancer Res..

[26] Bouta EM (2018). Targeting lymphatic function as a novel therapeutic intervention for rheumatoid arthritis. Nat. Rev. Rheumatol..

[27] Chen Y (2017). The pro-inflammatory cytokine TNF-α inhibits lymphatic pumping via activation of the NF-κB-iNOS signaling pathway. Microcirculation.

[28] Bouta EM (2013). Power Doppler ultrasound phenotyping of expanding versus collapsed popliteal lymph nodes in murine inflammatory arthritis. PLoS ONE.

[29] Rahimi H (2016). Lymphatic imaging to assess rheumatoid flare: mechanistic insights and biomarker potential. Arthritis Res. Ther..

[30] Manzo A (2016). Power Doppler ultrasonographic assessment of the joint-draining lymph node complex in rheumatoid arthritis: a prospective, proof-of-concept study on treatment with tumor necrosis factor inhibitors. Arthritis Res. Ther..

[31] Jung S (2000). Analysis of fractalkine receptor CX3CR1 function by targeted deletion and green fluorescent protein reporter gene insertion. Mol. Cell. Biol..

[32] Kikuta J (2013). Dynamic visualization of RANKL and Th17-mediated osteoclast function. J. Clin. Invest..

[33] Brand DD, Latham KA, Rosloniec EF (2007). Collagen-induced arthritis. Nat. Protoc..

[34] Kawamoto T (2003). Use of a new adhesive film for the preparation of multi-purpose fresh-frozen sections from hard tissues, whole-animals, insects and plants. Arch. Histol. Cytol..

[35] Xiao W (2016). Time-dependent gene expression analysis after mouse skeletal muscle contusion. J. Sport Heal. Sci..

