# Supplementary material for: Development of an intravital imaging system for the synovial tissue reveals the dynamics of CTLA-4 Ig in vivo
Source: Sci Rep. 2020 Aug 10;10:13480. doi: 10.1038/s41598-020-70488-y (PMC7417741; doi:10.1038/s41598-020-70488-y)
Supplement: Supplementary file 1 — Supplementary Information. [file 41598_2020_70488_MOESM1_ESM.pdf]

Development of an intravital imaging system for the synovial tissue reveals the dynamics of CTLA-4 Ig *in vivo*

Tetsuo Hasegawa[1,2], Junichi Kikuta[1,3], Takao Sudo[1], Erika Yamashita[1], Shigeto Seno[4], Tsutomu Takeuchi[2], Masaru Ishii[1,3]*

[1]Department of Immunology and Cell Biology, Graduate School of Medicine and Frontier Biosciences, Osaka University, Osaka, Japan
[2]Division of Rheumatology, Department of Internal Medicine, Keio University School of Medicine, Tokyo, Japan
[3]WPI-Immunology Frontier Research Center, Osaka University, Osaka, Japan
[4]Department of Bioinformatic Engineering, Graduate School of Information Science & Technology, Osaka University, Osaka, Japan

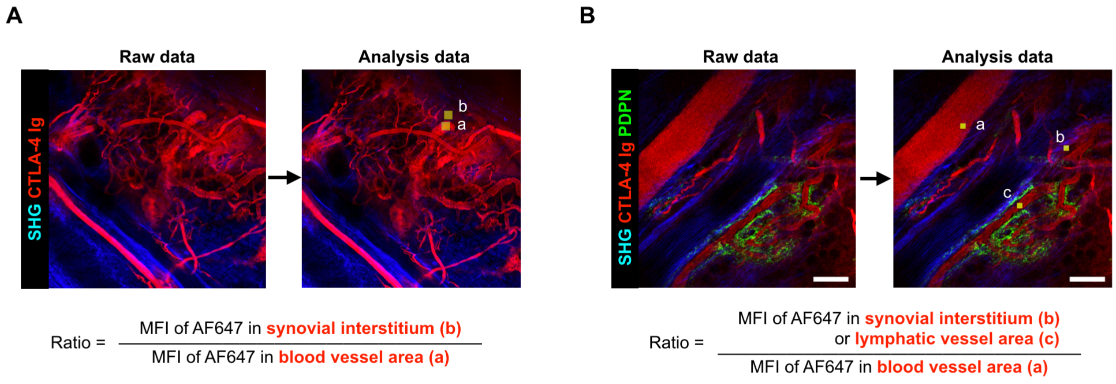

Supplementary Figure S1. Protocol for calculating permeability index.

(A, B) The blood vessel area, lymphatic vessel area, and interstitial area were manually circumscribed. The permeability index is defined as the ratio of mean fluorescence intensity (MFI) of AF647 in the synovial interstitial area or lymphatic vessel area to that in the blood vessel area in Figure 2C (A) and Figure 5C (B).

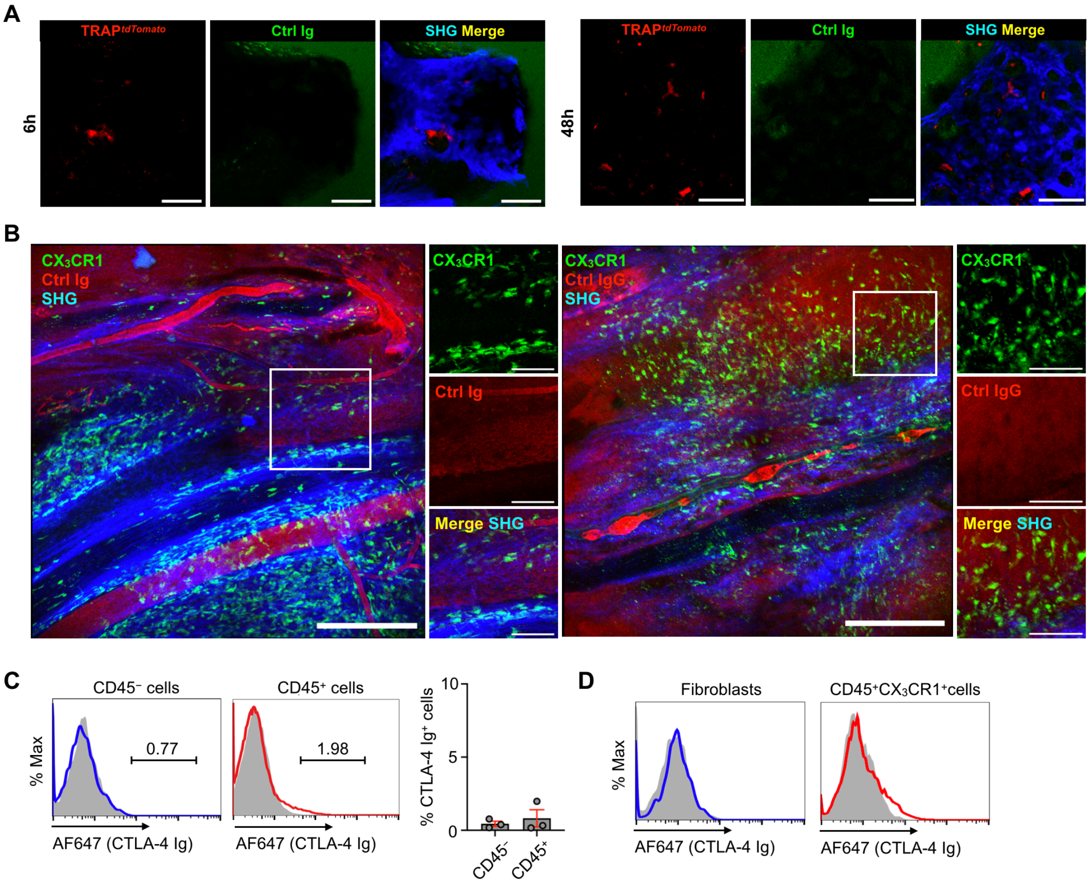

Supplementary Figure S2. Control IgG labelled with AF647 did not bind to osteoclasts, CX_3CR1^+ cells, and fibroblasts in the synovium.

(A) Intravital images of the third meta phalangeal joint of the CIA TRAP-tdTomato transgenic mice after injection of 200 μg of control IgG (AF647) at the indicated time points. Bars, 100 μm.

(B) Two intravital tile scan images of the CIA synovium of CX_3CR1 knock-in mice 3 hours after the injection with control IgG (AF647). Bars, 300 and 100 μm (high powered view).

(C) Histograms and cumulative data of control IgG $(AF647)^+$ cells among $CD45^+$ and $CD45^-$ cells in the inflamed synovium. n = 3 mice for each group. Shaded regions indicate cells from an untreated CIA mouse.

(D) Histogram plots of control IgG (AF647) fluorescence in fibroblasts and $CD45^+CX_3CR1^+$ cells in the inflamed synovium. Shaded regions indicate cells from an untreated CIA mouse.

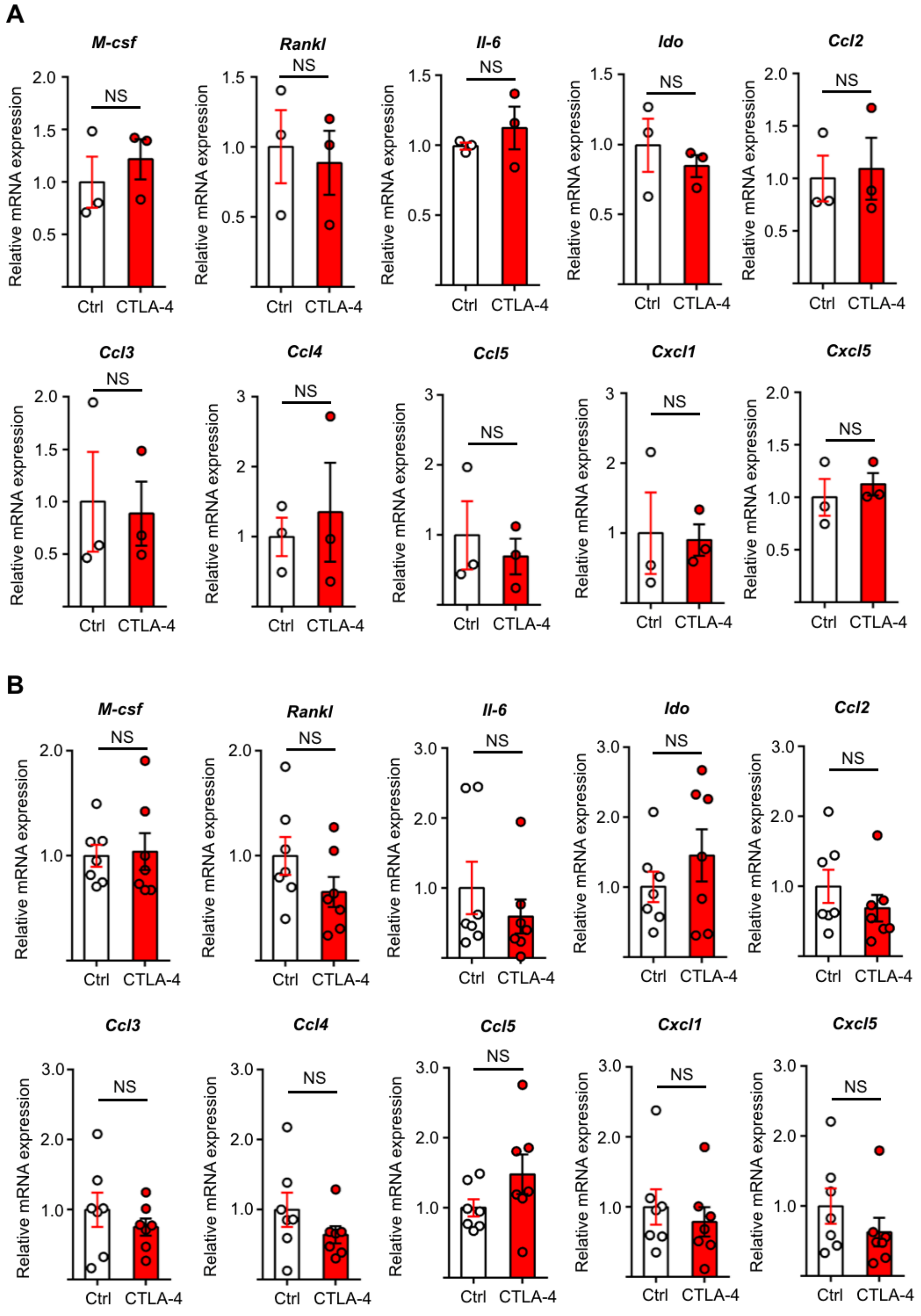

Supplementary Figure S3. RT-PCR analysis of synovial fibroblasts treated with CTLA-4 Ig.

(A) RT-PCR analysis of the expression of the indicated cytokines in synovial fibroblasts from CIA mice *in vitro*. Fibroblasts were sorted 1 week after the onset of arthritis and treated with 10 μg/ml CTLA-4 Ig or control IgG for 24 hours. *n* = 3 mice for each group.

(B) RT-PCR analysis of the expression of the indicated cytokines in synovial fibroblasts from CIA mice *in vivo*. 200 μg of CTLA-4 Ig or control IgG were injected intravenously 24 hours before sacrifice and FCM-sorted fibroblasts were analyzed. n = 7 mice for each group.

Unpaired two-tailed t test (A, B). Mean ± S.E.M. for each group.
